# A Modular Platform for Cytocompatible Hydrogels with Tailored Mechanical Properties Based on Monolithic Matrices and Particulate Building Blocks

**DOI:** 10.1021/acs.biomac.3c00177

**Published:** 2023-05-24

**Authors:** Lea Andrée, Pascal Bertsch, Rong Wang, Malin Becker, Jeroen Leijten, Peter Fischer, Fang Yang, Sander C. G. Leeuwenburgh

**Affiliations:** † Department of Dentistry—Regenerative Biomaterials, Radboud Institute for Molecular Life Sciences, Radboud University Medical Center, Philips van Leydenlaan 25, 6525 EX Nijmegen, The Netherlands; ‡ Department of Developmental BioEngineering, Faculty of Science and Technology, Technical Medical Centre, Leijten Laboratory, University of Twente, Drienerlolaan 5, 7522 NB Enschede, The Netherlands; § Department of Health Sciences and Technology, Institute for Food Nutrition and Health, ETH Zurich, Schmelzbergstrasse 7, 8092 Zurich, Switzerland

## Abstract

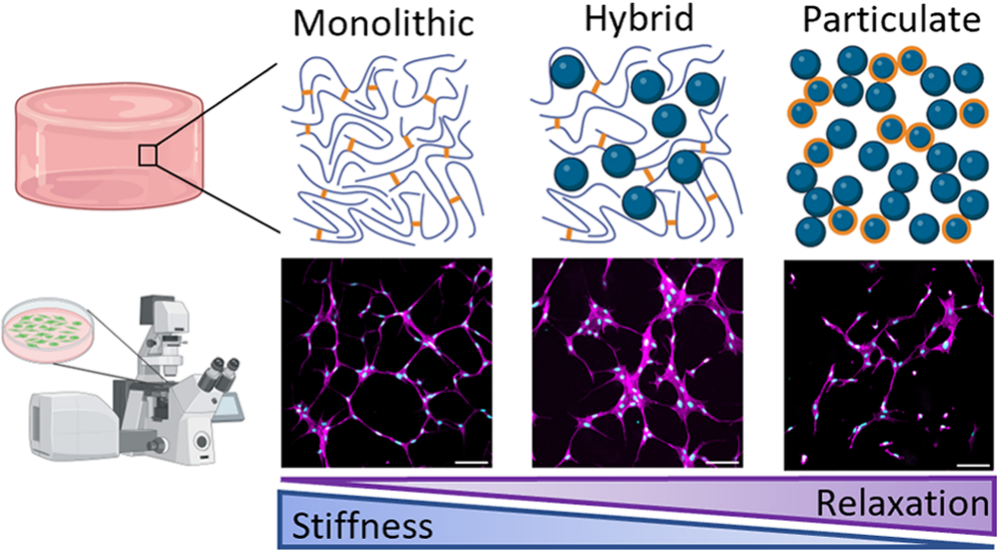

We establish a versatile hydrogel platform based on modular building blocks that allows the design of hydrogels with tailored physical architecture and mechanical properties. We demonstrate its versatility by assembling (i) a fully monolithic gelatin methacryloyl (Gel-MA) hydrogel, (ii) a hybrid hydrogel composed of 1:1 Gel-MA and gelatin nanoparticles, and (iii) a fully particulate hydrogel based on methacryloyl-modified gelatin nanoparticles. The hydrogels were formulated to exhibit the same solid content and comparable storage modulus but different stiffness and viscoelastic stress relaxation. The incorporation of particles resulted in softer hydrogels with enhanced stress relaxation. Murine osteoblastic cells cultured in two-dimensional (2D) on hydrogels showed proliferation and metabolic activity comparable to established collagen hydrogels. Furthermore, the osteoblastic cells showed a trend of increased cell numbers, cell expansion, and more defined protrusions on stiffer hydrogels. Hence, modular assembly allows the design of hydrogels with tailored mechanical properties and the potential to alter cell behavior.

## 1 Introduction

Hydrogels are widely used in biomedical engineering as injectable or implantable biomaterials, tissue engineering scaffolds, cell culture substrates, and three-dimensional (3D) (bio)printing inks.^1−4^ The mechanical properties of hydrogels have been recognized as a powerful cue to modulate cell behavior via mechanotransduction,^5,6^ and there have been increased efforts to design hydrogels that closely mimic the structure and mechanical behavior of the extracellular matrix (ECM) to optimize their cellular response. However, conventional monolithic hydrogels stabilized by covalent crosslinks usually exhibit limited potential to fine-tune their mechanical response, stressing the need for more dynamic and versatile hydrogel platforms.

The compressive stiffness of hydrogels, their resistance to elastic compressive deformation (Young’s modulus), has long been considered the main mechanical cue relevant for the mechanotransduction of ECM mimics. For differentiated cells, stiffer hydrogels generally induce more pronounced cell spreading and focal adhesion points and promote cell proliferation.^7,8^ For mesenchymal stromal/stem cells, soft hydrogels mimicking fat tissue favor adipogenic differentiation, while stiff hydrogels mimicking hard tissues favor osteogenic differentiation.^9−12^ More recently, it was recognized that the time-, strain-, and stress-dependent mechanical properties, i.e., viscoelasticity, of hydrogels also play an important role in mechanotransduction.^6^ Even hard skeletal tissues such as bone or cartilage are not fully elastic and exhibit viscoelastic stress relaxation upon deformation.^6,13^ Hydrogels with fast viscoelastic stress relaxation in hydrogels promote cell spreading, migration, and proliferation.^14−17^ Furthermore, fast-relaxing hydrogels trigger osteogenic differentiation of stem cells, while slow-relaxing hydrogels favor adipogenic differentiation.^18,19^ It is thus important to consider both stiffness and viscoelasticity when designing hydrogels for tissue regeneration, as already demonstrated by the enhanced in vivo regeneration of bone tissues using hydrogels with high stiffness and fast relaxation.^20,21^

Monolithic covalently crosslinked hydrogels generally exhibit a high stiffness and a purely elastic behavior, and thereby fail to mimic the viscoelasticity of the natural ECM. Hence, several strategies are currently being explored to create more complex and dynamic hydrogel systems by introducing reversible crosslinks,^22,23^ exploiting multiple crosslinking strategies (dual crosslinked hydrogels),^24,25^ or mixing of different polymers (double-network hydrogels).^25−27^ Furthermore, structurally more complex hydrogel building blocks such as fibrillar collagen,^28,29^ nano-^30−33^ or microparticles,^34,35^ ECM constituents,^36,37^ and combinations thereof^38−40^ are used to assemble hydrogels with more complex architecture and mechanical properties.

Gelatin is a popular base material for biomedical engineering as it is biocompatible, biodegradable, naturally contains cell-binding motifs, and can be readily modified or processed into different physical forms.^41^ Most commonly, gelatin is modified by methacrylic anhydride to obtain photo-crosslinkable gelatin methacryloyl (Gel-MA) hydrogels that are stable at body temperature. The formation of Gel-MA hydrogels at different Gel-MA concentrations is a straightforward approach to obtain hydrogels with different stiffness. However, Gel-MA hydrogels are primarily elastic due to their covalent crosslinks.^8,42,43^ Alternatively, gelatin can be processed into spherical gelatin nanoparticles (GNPs) that can be used to assemble particulate hydrogels.^30,31^ The GNPs may also be modified with methacryloyl groups to obtain photo-crosslinkable GNPs (GNP-MA).^44^ Such particulate hydrogels based on nanoparticles have the potential to provide a more dynamic environment for cell ingrowth as they are more viscoelastic than monolithic hydrogels.^32,38,45^ In addition, GNPs can be readily loaded with drugs and exploited for localized^46,47^ and intracellular delivery applications.^48−52^

Here, we establish a modular gelatin hydrogel platform based on monolithic matrices and particulate building blocks that allows the design of hydrogels with different physical architectures and mechanical properties. We showcase its applicability by assembling three model hydrogels, namely, (i) a monolithic Gel-MA hydrogel, (ii) a hybrid Gel-MA+GNPs hydrogel, and (iii) a purely particulate hydrogel composed of GNPs and GNPs-MA. The hydrogels were designed to have the same solid content and comparable storage moduli but different stiffness and viscoelastic stress relaxation to investigate their effect on cell activity and spreading in two-dimensional (2D) cell culture.

## 2 Materials and Methods

### 2.1 Gelatin Hydrogel Building Blocks

#### 2.1.1 Gelatin Methacryloyl

Gelatin methacryloyl (Gel-MA) obtained from type A gelatin (Bloom number 285) with a molecular weight of 160 kDa and a MA degree of substitution of 60% was provided by Rousselot BV.

#### 2.1.2 Gelatin Nanoparticle Synthesis

The same type A gelatin as used for Gel-MA production was provided as raw gelatin by Rousselot BV. GNPs were prepared by a desolvation process using acetone as described in detail before.^30^ In brief, 1.25 g of gelatin type A was dissolved in 25 mL of demineralized water under stirring at 40 °C. The pH was lowered to 2.5 using 6 M HCl (37% fuming, Merck), whereafter 60 mL of acetone (Boom) was added dropwise (8 mL/min) under stirring to induce gelatin desolvation and aggregation into spherical GNPs. The GNPs were crosslinked by the addition of 316 μL of 25 wt % glutaraldehyde solution (Acros) under stirring at room temperature. After 16 h, the crosslinking was stopped by neutralization of glutaraldehyde using 100 mL of 100 mM glycine (Sigma) solution. The GNPs were washed two times by centrifugation and redispersion in demineralized water, and washed GNPs were stored in demineralized water at 4 °C. A Malvern Zetasizer Nano-Z dynamic light scattering device was used to determine GNP hydrodynamic diameter dispersed in demineralized water and GNP ζ-potential dispersed in 5 mM HEPES buffer (Sigma) at pH 7.4. The obtained GNPs had a hydrodynamic diameter of 480.4 ± 4.2 nm with a polydispersity index of 0.04 ± 0.02 and a ζ-potential of +14.55 ± 0.14 mV.

#### 2.1.3 Gelatin Nanoparticle Modification with Methacryloyl Groups

GNPs were prepared by desolvation as described above. To obtain smaller GNPs prior to modification, the pH was adjusted to pH 3, and ethanol (180 mL) was used as nonsolvent instead of acetone. A crossflow setup based on a Sartorius Stedim Sartocon Slice filter holder equipped with a 300 kDa cutoff membrane was used to remove ethanol and wash GNPs. The obtained GNPs had an average hydrodynamic diameter of 274.6 ± 2.4 nm with a polydispersity index of 0.09 ± 0.02 and a ζ-potential of +23.8 ± 0.7 mV. The GNPs were functionalized with methacryloyl groups directly after preparation by dispersion in 1 M carbonate-bicarbonate buffer (CB, 0.09 M sodium carbonate and 0.91 M sodium bicarbonate, Merck) and demineralized water to reach nanoparticle and CB buffer concentrations of 10 mg/mL and 0.1 M, respectively. The solution was heated to 50°C, and pH was adjusted to 9 by addition of a 1 M NaOH (Merck) solution under stirring. Methacrylic anhydride (MA, Sigma) was added dropwise to the mixture at a concentration of 1.16 mL MA/g GNPs. The solution was kept at pH 9 during addition of MA. After 1 h at 50 °C, the GNP-MA dispersion was washed three times with 1 L of demineralized water using crossflow filtration to remove unreacted MA. The modified GNPs-MA had an average hydrodynamic diameter of 294.5 ± 8.7 nm with a polydispersity index of 0.12 ± 0.05 and a ζ-potential of −31.6 ± 0.3 mV. The crosslinking degree of GNPs was determined via the amine content of GNPs by a modified colorimetric 2,4,6-trinitrobenzene sulfonic acid (TNBS, Sigma) assay.^53^ After desolvation the GNPs exhibited an amine consumption of 28.1% relative to raw gelatin due to crosslinking with glutaraldehyde. The final GNPs-MA had an amine consumption of 91.3%, indicating a degree of MA substitution of ≈63% comparable to the used Gel-MA.

### 2.2 Hydrogel Preparation and Characterization

#### 2.2.1 Hydrogel Preparation

**Figure 1:**
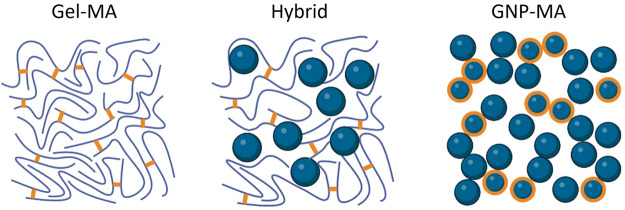
Schematic of the physical architecture of the three gelatin-based hydrogels. Left: Fully monolithic Gel-MA with photo-crosslinked MA-groups indicated in orange. Middle: Hybrid hydrogel based on 1:1 Gel-MA and particulate GNPs. Right: Fully particulate hydrogels based on 1:2 GNPs-MA and GNPs.

Fully monolithic Gel-MA hydrogels were prepared by dissolving 6 wt/v% Gel-MA at 37 °C in PBS containing 0.1% lithium-phenyl-2,4,6-trimethylbenzoylphosphinate (LAP, Sigma) as photoinitiator and subsequent photo-crosslinking for 2 min using a 5 W Cree Ultrafire S5 405 nm blue light lamp. Hybrid hydrogels were also formed at a total gelatin solid content of 6 wt/v% from equal amounts (1:1) of Gel-MA and unmodified GNPs obtained by desolvation using acetone (*d* = 480.4 nm, +14.55 mV). The GNPs stored in demineralized water were collected by centrifugation at 21,000 rcf for 20 min and mixed with Gel-MA dissolved in PBS at 37 °C containing a final LAP concentration of 0.1%, whereafter the hybrid hydrogels were photo-crosslinked as described above. Fully particulate hydrogels were prepared from 1:2 GNPs-MA (*d* = 294.5 ± 8.7 nm, −31.6 ± 0.3 mV) and unmodified GNPs obtained by desolvation using ethanol (*d* = 274.6 ± 2.4 nm, +23.8 ± 0.7 mV) to obtain a charge-neutral dispersion. Particle dispersions with neutral charge were crucial to achieve maximum *G*′, as further visualized in Figure S1C. The two countercharged particles stored in demineralized water were mixed together and let to interact for 30 min before addition of 0.1% LAP and collection of the particles by centrifugation at 1500 rcf for 15 min prior to photo-crosslinking as described above. Collagen hydrogels were prepared as controls from type I rat tail collagen (BD Biosciences, 4.08 mg/mL in 0.2 M acetic acid) as instructed by the manufacturer by mixing 75 v% collagen stock solution with 13 v% demineralized water, 10 v% 10× PBS, and 2 v% 1 M NaOH on ice to obtain a 0.3 wt/v% collagen solution with pH 7.4 that was thermogelled in an incubator at 37 °C for 1 h. A schematic of the physical architecture of the three gelatin-based hydrogels is shown in Figure 1.

#### 2.2.2 Gelation Kinetics

The gelation kinetics of photo-crosslinkable hydrogels (Gel-MA, Hybrid, GNP-MA) were determined by an Anton Paar MCR 702 rheometer with a bottom glass plate with temperature control and the blue light lamp (5 W, 405 nm) mounted underneath. The hydrogel precursors were loaded on the glass plate and the upper plate geometry was lowered to a gap size of 400 μm and sealed with silicon oil. A 25 mm crosshatched (Gel-MA, Hybrid) or sandblasted (GNP-MA) upper geometry was used to avoid material slip. A time sweep was started at a constant frequency of 1 rad/s and strain amplitude of 1% at 37 °C, and the photo-crosslinking was started after 1 min. The thermogelation of collagen hydrogels was determined using a TA AR2000ex rheometer. The hydrogel precursor was loaded on the bottom plate at 4 °C, whereafter an upper crosshatched 20 mm plate was lowered to 400 μm and sealed with silicon oil. A time sweep was started at a constant frequency of 1 rad/s and strain amplitude of 1% at 4 °C, and the temperature was increased to 37 °C after 1 min.

#### 2.2.3 Strain Sweep and Recovery

Strain sweeps were performed after the gelation experiments in a strain range from 0.1 to 1000% at a constant frequency of 1 rad/s at 37 °C. The recovery of the hydrogels after high strains was determined by a time sweep following the strain sweep at a constant frequency of 1 rad/s and strain amplitude of 1%. Experiments were performed after photo-crosslinking (gelatin-based hydrogels) or thermogelation (collagen) at 37 °C.

#### 2.2.4 Stress Relaxation

The stress relaxation of hydrogels was determined in step-strain experiments using a previously established protocol that allows stress relaxation measurements at stepwise increasing strain on the same hydrogel sample.^45^ In brief, a step-strain was performed within a strain-raise time of 0.2 s, whereafter the strain was kept constant for 10 min to measure relaxation. After the relaxation phase, the geometry was slowly turned back (negative strain) until stress was 0. After a 10 min equilibration phase, the next step-strain at higher strain was performed. This protocol allowed measurement of stress relaxation at stepwise increasing strain on the same sample without cumulative stress buildup, given that the hydrogels are self-healing at the tested strains. To facilitate comparison of stress relaxation for different hydrogels and strains, stress relaxation is also depicted as normalized stress, normalized by the individual maximum stress. Experiments were performed after photo-crosslinking (gelatin-based hydrogels) or thermogelation (collagen) at 37 °C.

#### 2.2.5 Stiffness Measurement

The compressive stiffness, i.e., Young’s modulus, of hydrogels was determined in uniaxial compression experiments using an Optics11 Life Pavone nanoindenter equipped with a spherical tip with 24.5 μm radius and 0.26 N/m stiffness. Hydrogels were prepared in round Teflon molds that were removed after 2 min photo-crosslinking and samples were stored in 1× PBS at 4 °C overnight. To assess local mechanical properties hydrogels were indented to a depth of 2 μm in PBS at 22 °C. 36 indentation curves were obtained per hydrogel condition on multiple hydrogels and positions within the same hydrogel and fitted with the Hertzian contact model to determine Young’s moduli, assuming a Poisson ratio of 0.5. The number of acceptable fits (*R*^2^ ≥ 0.95) was *n* = 36 for Gel-MA and *n* = 32 for Hybrid and GNP-MA hydrogels. Due to extremely low stiffness values, the indentation depth was increased to 3.9 μm and fits with *R*^2^ ≥ 0.9 were accepted (*n* = 22) for collagen hydrogels.

#### 2.2.6 Hydrogel Swelling Experiment

Hydrogel swelling was quantified by gelling 50 μL of hydrogels (*n* = 3) and covering them with PBS and gravimetric measurement of swelling after storage for 3 days at 4 °C.

### 2.3 Hydrogel-Based Cell Culture

#### 2.3.1 Cell Culture

Subconfluent culture of murine pre-osteoblasts of the cell line MC3T3-E1 subclone 4 (CRL-2593, American Type Culture Collection) was maintained in Minimal Essential Medium α (Gibco, MEM-α; Catalog number: A22571-020), supplemented with 10% FBS (Gibco; Catalog number: 10270-106) and 100 units/mL penicillin and 0.1 mg/mL streptomycin (Sigma-Aldrich). This pre-osteoblastic cell line was selected since the hydrogels studied herein are envisioned for application in bone regeneration.

#### 2.3.2 Cell Metabolic Activity Assay

50 μL of hydrogel was pipetted in a 96-well plate (Greiner), photo-crosslinked for 2 min, and incubated with full culture medium for 1 h at 37 °C and 5% CO_2_. The medium was removed, and cells were seeded at 7500 cells/cm^2^ in 200 μL of full culture medium. The cells were kept in an incubator at 37 °C and 5% CO_2_. Cell viability was assessed by measuring metabolic activity using the cell counting kit 8 (CCK-8) assay after 1, 3, and 7 days of culture. A stock solution of 5 mM water-soluble tetrazolium 8 (WST-8, 5-(2,4-disulfophenyl)-3-(2-methoxy-4-nitrophenyl)-2-(4-nitrophenyl)-2*H*-tetrazolium, inner salt, monosodium salt, Cayman Chemicals) and 0.2 mM 1-methoxy-5-methylphenazinium (TCI Chemicals) was prepared in 150 mM sodium chloride (Merck) and stored at -80°C until further use. After 1, 3, and 7 days of incubation, the medium was replaced by 200 μL of full culture medium containing 10 v/v% CCK-8 solution and incubated for 3.5 h at 37 °C. The absorbance of 100 μL of medium was measured in a new 96-well plate at 460 and 650 nm (machine background) on a spectrophotometer (Synergy HTX multimode reader, Biotek). For measurement of metabolic activity after 7 days, the culture medium was replaced with 200 mL of fresh full culture medium at day 3.

#### 2.3.3 Cell Imaging

10 μL of hydrogels was pipetted in a μ-slide angiogenesis (ibidi), photo-crosslinked for 2 min as described above, and incubated with full culture medium for 1 h at 37 °C. The medium was removed, and cells were seeded at 3000 cells/cm^2^ in 50 μL of full culture medium. After 3 days, samples were washed thrice with PBS (Gibco) and fixed with 4% formaldehyde (Sigma) for 15 min at room temperature (RT), followed by washing three times with PBS. Cells were permeabilized using 0.5 v/v% Triton X-100 (Sigma) in PBS for 10 min at RT and washed thrice with PBS. Actin filaments were stained with phalloidin AlexaFluor-568 conjugate (Invitrogen) in PBS for 30 min at RT protected from light, followed by washing thrice with PBS. For cells seeded on Hybrid and GNP-MA hydrogels, a dilution of 1:20 was used, whereas a dilution of 1:50 and 1:100 was used for Gel-MA, collagen, and tissue culture plastic (TCP), respectively. Nuclei were stained with Hoechst 33342 (Invitrogen) in PBS for 30 min at RT protected from light, followed by washing thrice with PBS. For cells seeded on Hybrid and GNP-MA hydrogels, a concentration of 200 μg/mL was used, whereas a concentration of 20 and 2 μg/mL was used for Gel-MA, collagen, and TCP, respectively. Samples were stored at 4 °C until image acquisition with an LSM900 confocal microscope (Zeiss). Phalloidin AlexaFluor-568 conjugate was excited at 561 nm (emission filter: 575–700 nm) and Hoechst was excited at 405 nm (emission filter: 415–575 nm). The number of cells was determined by nuclei count on three to five confocal images using Fiji.^54^ The signal intensity of images was adjusted using the maximum filter (radius 5 pixel), and nuclei were counted automatically by finding intensity maxima (point selection, prominence >80). A representative image of each hydrogel condition is shown in Figure S2.

#### 2.3.4 Statistical Analysis

Statistical analysis was performed with Prism version 8.4 (GraphPad). Outliers were identified by robust regression and outlier removal (ROUT method) with *Q* = 2% and removed prior to statistical analysis. Cell metabolic activity data was tested for normality using a Shapiro–Wilk test and analyzed by two-way analysis of variance (ANOVA) with Tukey multiple comparison correction to detect differences between the different hydrogel groups. Experiments were performed in sextuplicate, and data was pooled over two experiments (*n* = 12). Cell number data obtained from confocal images (*n* = 3–5) was analyzed using Brown-Forsythe and Welch ANOVA with Dunnet’s multiple comparison correction. All data are presented as mean ± standard deviation. Significance was set at *p* < 0.05 and *p* values are reported using *p < 0.05, **p < 0.01, ***p < 0.001, and ****p < 0.0001.

## 3 Results and Discussion

### 3.1 Hydrogel Design and Composition

Based on the three gelatin structural components Gel-MA, GNPs, and GNP-MA, we designed three modular hydrogels with different physical architectures: (i) a fully monolithic photo-crosslinked Gel-MA hydrogel, (ii) a hybrid hydrogel consisting of equal amounts of Gel-MA and GNPs, and (iii) a fully particulate hydrogel consisting of GNPs-MA and GNPs (weight ratio of 1:2). An overview of their composition and mechanical properties is provided in Table 1 and a schematic of their physical architecture is depicted in Figure 1. The solid content of all three types of hydrogels was fixed at 6 wt/v%. The three hydrogels exhibited similar storage moduli *G*′ ≈ 1200–1500 Pa after photo-crosslinking but different local stiffness, i.e., Young’s moduli, in uniaxial compression. To evaluate the performance of the hydrogels in cell culture, 0.3 wt/v% collagen I hydrogels, which are widely employed in hydrogel cell culture,^4^ were used as a control. The mesh size of swollen Gel-MA hydrogels is in the range of tens of nm^55^ while the incorporated GNPs are 480 nm in diameter. We thus expect that the incorporated particles can impair the interconnectivity of the Gel-MA network in Hybrid hydrogels. The photo-gelation and rheology of other modular hydrogel formulations are discussed as Supporting Information (Figure S1).

**Table 1:** Overview of the Three Gelatin-Based Hydrogel Systems and Reference Collagen Showing Their Physical Architecture, Composition, Solid Content (wt/v%), Storage Modulus *G*′ after Crosslinking at 1% Strain and 1 rad/s, and Stiffness (Young’s Modulus) in Uniaxial Compression

hydrogel	architecture	composition	solid content (wt/v%)	storage modulus *G*′ (γ = 1%, ω = 1 rad/s) (Pa)	stiffness (Young’s modulus) (kPa)
Gel-MA	monolithic	Gel-MA	6	1462 ± 151	4.95 ± 0.54
Hybrid	hybrid	1:1 Gel-MA + GNPs	6	1267 ± 246	1.43 ± 0.65
GNP-MA	particulate	1:2 GNPs-MA + GNPs	6	1205 ± 93	0.60 ± 0.41
collagen	fibrous	collagen	0.3	18 ± 0.1	0.03 ± 0.01

### 3.2 Hydrogel Mechanical Characterization

#### 3.2.1 Gelatin Hydrogel Photo-Gelation and Rheology

**Figure 2:**
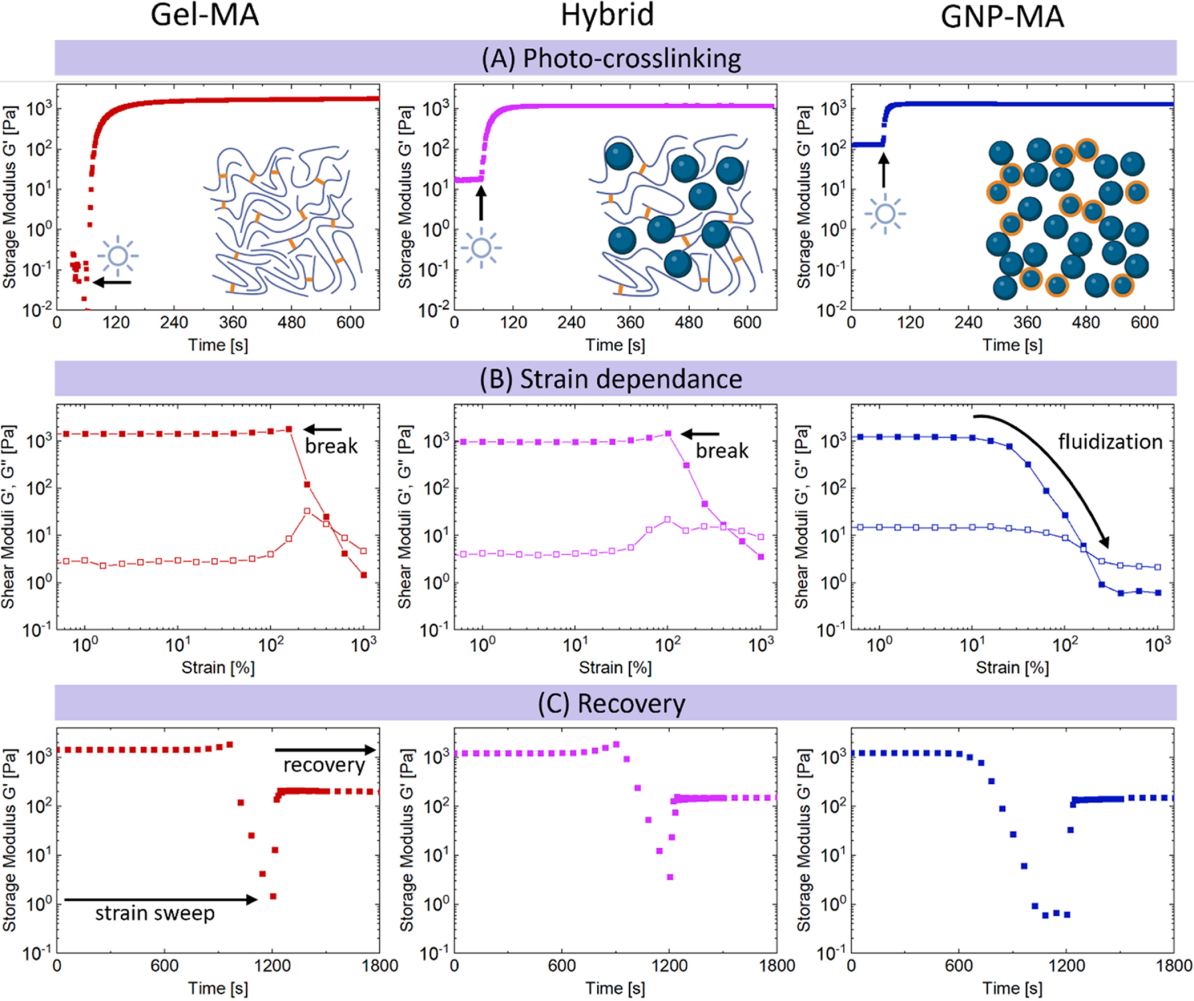
Rheological characterization of 6 wt/v% gelatin-based hydrogels showing (A) their gelation kinetics upon exposure to blue light expressed by increase in dynamic storage modulus *G*′, (B) their strain dependence in strain sweeps showing storage modulus *G*′ (full) and loss modulus *G*″ (empty), and (C) their recovery of *G*′ after strain sweeps. Experiments were performed at 37 °C at 1% strain and a frequency of 1 rad/s. Strain sweeps were performed from 0.1 to 1000% strain.

All hydrogel precursors could be pipetted and molded prior to photo-crosslinking. To investigate their gelation kinetics and viscoelasticity after photo-crosslinking, their transient rheology upon exposure to blue light was measured using a rheo-optics setup with a transparent bottom plate. Figure 2A depicts the evolution of the dynamic storage modulus *G*′ of the three gelatin hydrogels upon exposure to blue light. The initial *G*′ prior to photo-crosslinking increased with increasing amount of GNPs, in the order Gel-MA < Hybrid < GNP-MA. Upon photo-crosslinking *G*′ increased and equilibrated at ≈1200–1500 Pa for all hydrogels after 1–2 min. Comparable gelation kinetics and *G*′ were previously reported for Gel-MA^56^ and GNP-MA^44^ hydrogels. Hence, despite their different hydrogel architecture, all three hydrogels exhibited similar rheological characteristics at small strains, i.e., at rest or small deformations. However, the specific hydrogel architecture greatly affected the rheological behavior of the hydrogels at increasing strain, as visualized by strain sweeps in Figure 2B. Gel-MA and Hybrid hydrogels showed a broad linear viscoelastic regime with constant moduli up to 100% strain followed by a brittle breakage. The Hybrid hydrogel showed a strain dependence almost identical to the monolithic Gel-MA, despite the fact that this gel contained 50% nanoparticles. This observation indicates that the rheological behavior of Hybrid hydrogels remained dominated by the Gel-MA matrix, whereas embedded GNPs did not affect its rheology considerably. On the other hand, the particulate GNP-MA hydrogels showed gradual fluidization and decrease in *G*′ above ≈10% strain. We have previously noted this difference in strain dependence between monolithic hydrogels (brittle breakage) and particulate hydrogels made of unmodified GNPs (fluidization).^45^ This strain-dependent behavior of particulate hydrogels is thus preserved after introducing one-third of photo-crosslinkable GNP-MA. The strain dependence of GNP-MA hydrogels could be particularly valuable as the hydrogel fluidizes at 10–50% strain, which are strains typically associated with cell activity,^57,58^ and fluidization in this strain range is a predictor of accelerated stress relaxation,^45^ as discussed in detail below.

Figure 2C shows the strain sweeps from Figure 2B plotted as a function of time followed by oscillation at small strains to test the recovery of hydrogels after strain sweeps. All three gelatin hydrogels showed a limited recovery after strain sweeps, indicating that the hydrogels are not dynamic enough to be self-healing and recover from large strains due to covalent MA-crosslinks.^1,59^ The particulate GNP-MA hydrogel also lost its self-healing capacity as opposed to hydrogels composed of unmodified GNPs,^30,45^ although only one-third of the embedded particles were modified with photo-crosslinkable methacryloyl groups.

#### 3.2.2 Gelatin Hydrogel Viscoelastic Stress Relaxation

**Figure 3:**
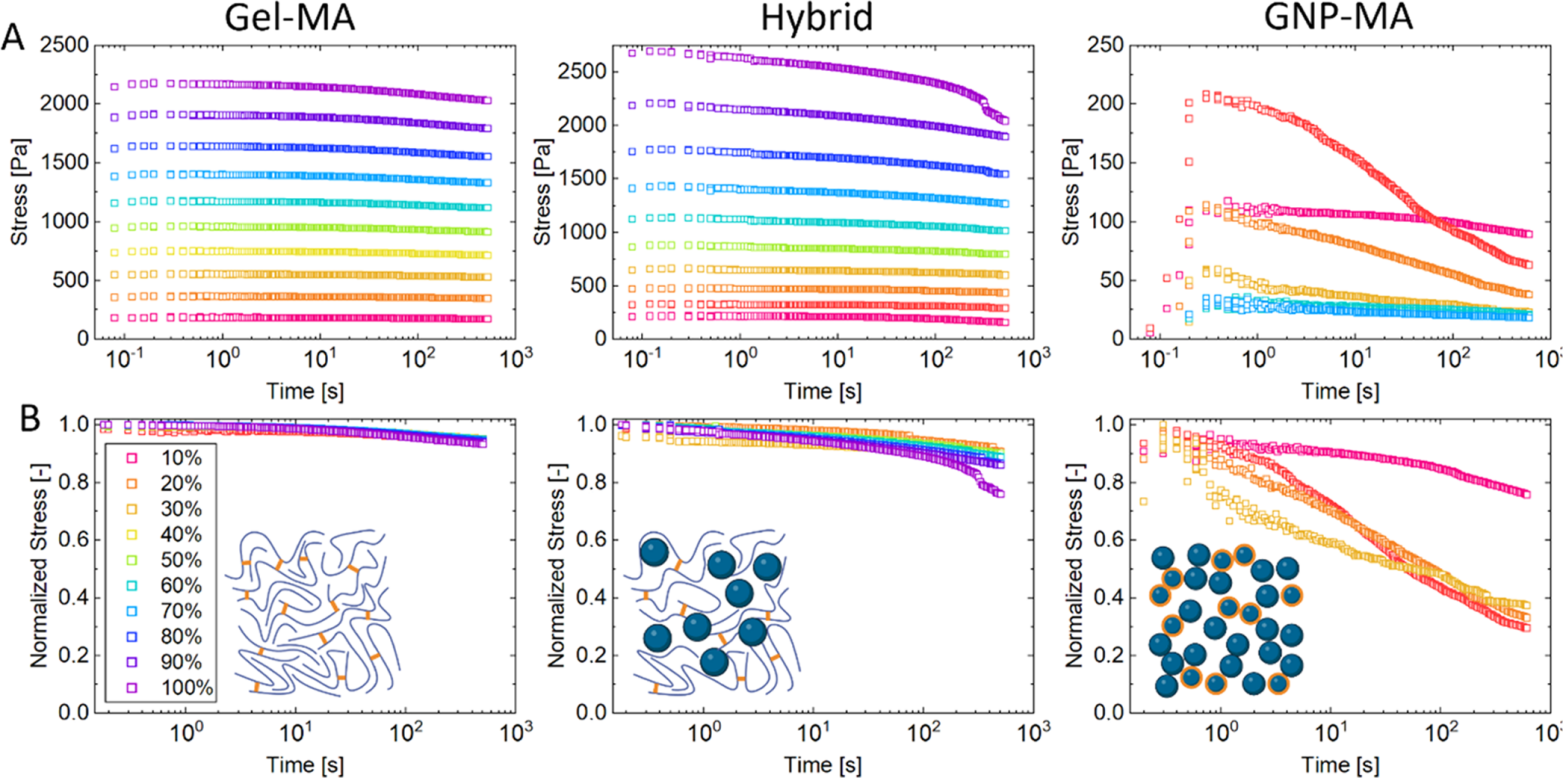
Viscoelastic stress relaxation of different gelatin-based hydrogels at increasing strain expressed as (A) raw stress during step-strain experiments (0.2 s strain raise followed by 10 min relaxation at constant strain) and (B) normalized by the maximum stress.

Covalently crosslinked hydrogels are often primarily elastic and fail to mimic the viscoelastic stress relaxation of the natural ECM. The design of hydrogels based partially or entirely on particles is an emerging approach to obtain more viscoelastic hydrogels.^38,42,45^Figure 3 shows the viscoelastic stress relaxation of the three gelatin-based hydrogel systems at stepwise increasing strains from 10 to 100% expressed as (A) raw stress and (B) normalized by the individual stress peak. For the monolithic Gel-MA and Hybrid hydrogels, the induced raw stress increased linearly with strain, indicating a primarily elastic behavior following Hooke’s law. The stress relaxation of Gel-MA hydrogels was limited to ≈7% within 10 min for all strains with no major strain dependence. This very limited stress relaxation of Gel-MA hydrogels was expected in view of the irreversible nature of the covalent crosslinks. The Hybrid hydrogel with 50% incorporated GNPs exhibited a slightly increased stress relaxation of 10–15% with a tendency to increase relaxation at higher strain. We have previously found that hydrogels based entirely on unmodified GNPs show fast exponential stress relaxation in this strain range.^45^ Hence, despite the incorporation of 50% of these GNPs by solid content the resulting Hybrid hydrogel showed limited stress relaxation and was primarily dominated by the covalent Gel-MA matrix. Even for Hybrid hydrogel formulations based on higher particle fractions, i.e., 1:2 Gel-MA + GNPs, the stress relaxation was only slightly enhanced to ≈20% as shown in Figure S2. It is important to note that rheology captures the macroscopic hydrogel properties. We speculate that the incorporation of GNPs may still alter the mechanical properties at smaller, i.e., cellular scale, such as in nanoindentation as discussed below. On the other hand, fully particulate GNP-MA hydrogels showed a much lower raw stress buildup and accelerated stress relaxation of 20% at 10% strain and even 70% relaxation at 20% strain. Due to the fluidization of the GNP-MA hydrogel at higher strains and limited self-healing capacity of the GNP-MA hydrogel (see Figure 2B,C), stress measurements were impaired at further increasing strains. Hence, the accelerated stress relaxation of particulate hydrogels was preserved upon incorporation of photo-crosslinkable GNPs-MA, although stress relaxation was slower compared to fully particulate hydrogels composed of unmodified GNPs.^45^ This divergent stress relaxation behavior of different hydrogels is in line with their strain dependence previously discussed in Figure 2B. While Gel-MA and Hybrid hydrogels were linear elastic up to 100% strain, the GNP-MA hydrogels showed steady fluidization at <10% strain. This confirms our previous observation that stress relaxation in hydrogels is accelerated at strains beyond the linear viscoelastic regime where hydrogel network rearrangements occur, and strain sweeps thus are a good predictor for strain-dependent stress relaxation.^45^ Gel-MA and Hybrid hydrogels may also exhibit accelerated stress relaxation at strains >100%, as apparent from the sudden stress drop for Hybrid hydrogels at 100% strain. However, from the present hydrogels, only the fully particulate GNP-MA hydrogels are expected to exhibit an accelerated stress relaxation at strains 10–50%, which are relevant for cell activity.^57,58^

#### 3.2.3 Collagen Hydrogel Rheology and Viscoelastic Stress Relaxation

**Figure 4:**
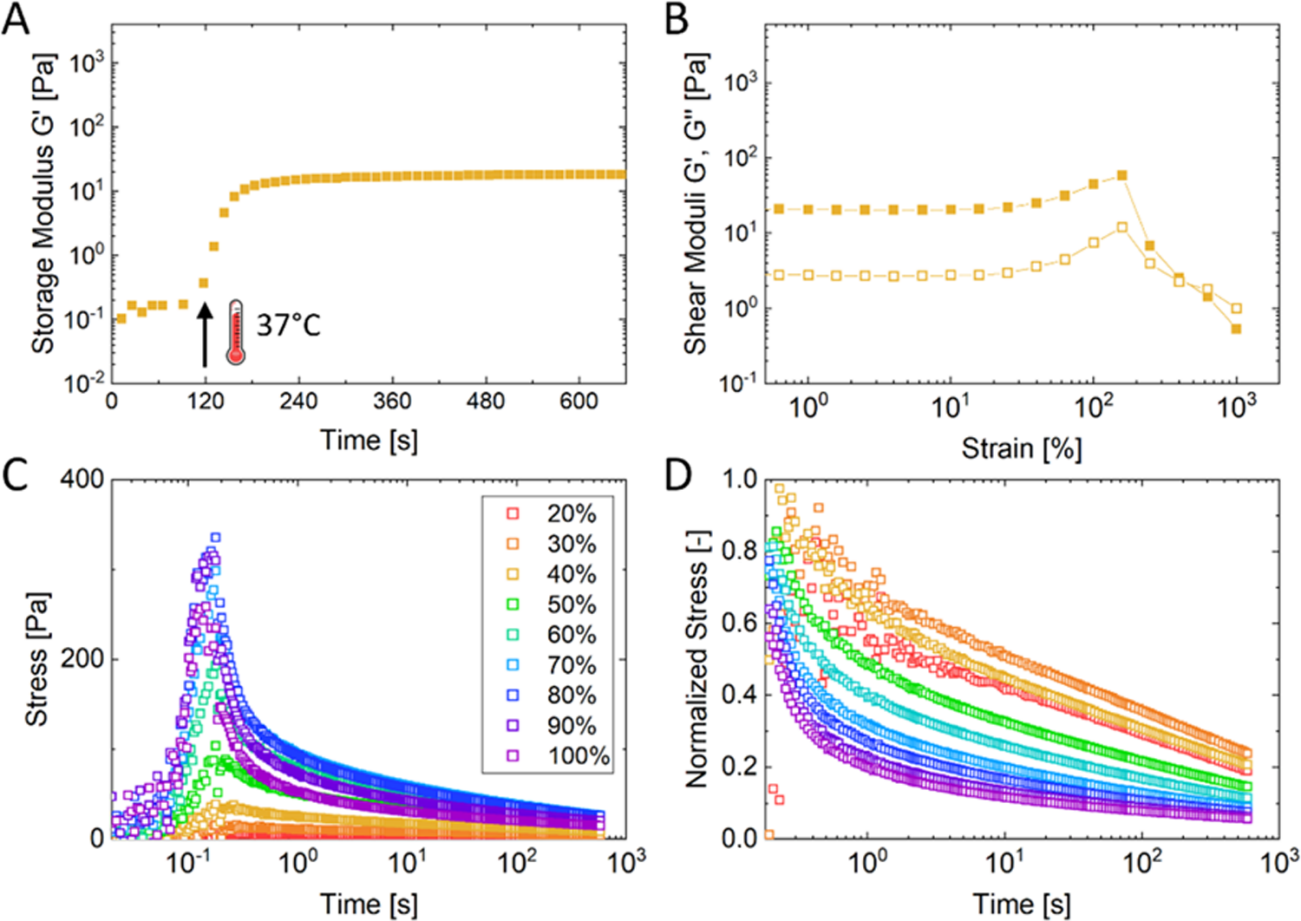
Rheological characterization of 0.3 wt/v% collagen hydrogels showing (A) their gelation kinetics upon heating from 4 to 37 °C expressed by increase in dynamic storage modulus *G*′ and (B) their strain dependence in strain sweeps showing storage modulus *G*′ (full) and loss modulus *G*″ (empty). (C) Viscoelastic stress relaxation at increasing strain expressed as raw stress during step-strain experiments (0.2 s strain raise followed by 10 min relaxation at constant strain) and (D) normalized by the maximum stress.

Collagen hydrogels are among the most widely used hydrogels for cell culture^4^ and were used as a control to assess the performance of gelatin-based hydrogels in cell culture. To facilitate this comparison with the above-described gelatin-based hydrogels, collagen hydrogels were rheologically characterized using the same procedures. Figure 4A shows the thermogelation of collagen upon heating from 4 to 37 °C. The collagen hydrogels thermogelled within 2 min and equilibrated at *G*′ ≈ 18 Pa, which was considerably lower than the storage moduli of gelatin-based hydrogels (*G*′ ≈ 1200–1500 Pa, see Figure 2A) due to the much lower solid content. In strain sweeps (Figure 4B), the collagen hydrogels showed a linear viscoelastic response up to a strain of ≈20% followed by increasing *G*′ and *G*″ denoting strain stiffening before breakage at >100% strain. Hence, collagen hydrogels show a distinct strain stiffening at strains relevant for cell activity as previously reported.^60−62^Figure 4C,D shows the stress relaxation of collagen hydrogels at strains from 20 to 100% (no stress relaxation could be measured at 10% strain as the induced raw stress was too low) expressed as raw stress and normalized stress, respectively. At low strains (20–40%) the induced raw stress scaled linearly with strain and the stress relaxation was linear. At higher strains, the induced raw stress was disproportionately high due to the strain stiffening behavior, but the stress relaxation was considerably faster and exponential in this strain range. The accelerated stress relaxation of collagen hydrogels at increasing strain was previously reported elsewhere.^63^ Hence, collagen hydrogels stiffen at strains induced by cells, but the induced stress is relaxed quickly at the time scale of seconds. Compared to the gelatin-based hydrogels investigated here, collagen hydrogels exhibited a faster and higher extent of stress relaxation.

#### 3.2.4 Hydrogel Compressive Stiffness

**Figure 5:**
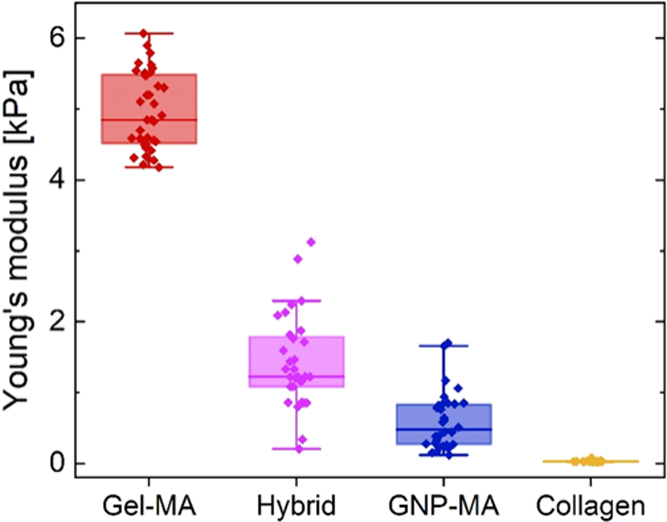
Stiffness (Young’s modulus) distribution of gelatin-based and collagen hydrogels obtained from nanoindentation uniaxial compression experiments.

Figure 5 shows the Young’s modulus distribution of gelatin-based and control collagen hydrogels. Monolithic Gel-MA hydrogels were considerably stiffer (4.95 ± 0.54 kPa) compared to Hybrid (1.43 ± 0.65 kPa), GNP-MA (0.60 ± 0.41 kPa), and collagen (0.03 ± 0.01 kPa) hydrogels. Although all three gelatin-based hydrogels exhibited similar storage moduli in oscillatory rheology (*G*′ ≈ 1200–1500 Pa, Figure 2) their compressive stiffness in uniaxial compression using nanoindentation deviated up to 8-fold. This is a significant observation given that storage modulus *G*′ and stiffness are sometimes used synonymously in the mechanical characterization of hydrogels. For homogeneous linear elastic hydrogels, it is expected that the Young’s modulus *E* and shear modulus *G* are correlated according to *E* = 2*G*(1 + ν), where ν is Poisson’s ratio = 0.5. This correlation was indeed met for monolithic Gel-MA hydrogels, but not for heterogeneous Hybrid or fully particulate GNP-MA hydrogels. Hence, the shear moduli and stiffness (Young’s modulus) may be decoupled for hydrogels with more complex architecture beyond monolithic covalently crosslinked hydrogels, and apparent mechanical properties of structurally complex hydrogels may depend on the mode and length scale of deformation, i.e., macroscopic shear rheology vs. local uniaxial compression. While rheology applies a rotational strain to a macroscopic hydrogel sample, nanoindentation applies a localized uniaxial compression using a micron-sized tip, which might be more sensitive in measuring differences in crosslinking density or dissipative structures such as freely movable GNPs. Furthermore, it is likely that hydrogels containing particles are more compressible than fully elastic hydrogels and exhibit a Poisson’s ratio <0.5. In contrast to rheological experiments, nanoindentation was performed in liquid to avoid sample drying and probe sticking, potentially leading to hydrogel swelling and decreased stiffness. However, the present hydrogels did not swell excessively in PBS, namely, 0.8 ± 0.4% for Gel-MA, 1.5 ± 0.1% for Hybrid, 3.7 ± 0.2% for GNP-MA, and 1.4 ± 0.7% for collagen hydrogels. Furthermore, measurements in liquid represent the state of hydrogels during cell culture and provide the closest resemblance to the mechanical hydrogel environment perceived by cells growing in 2D culture.

### 3.3 Hydrogel 2D Cell Culture

**Figure 6:**
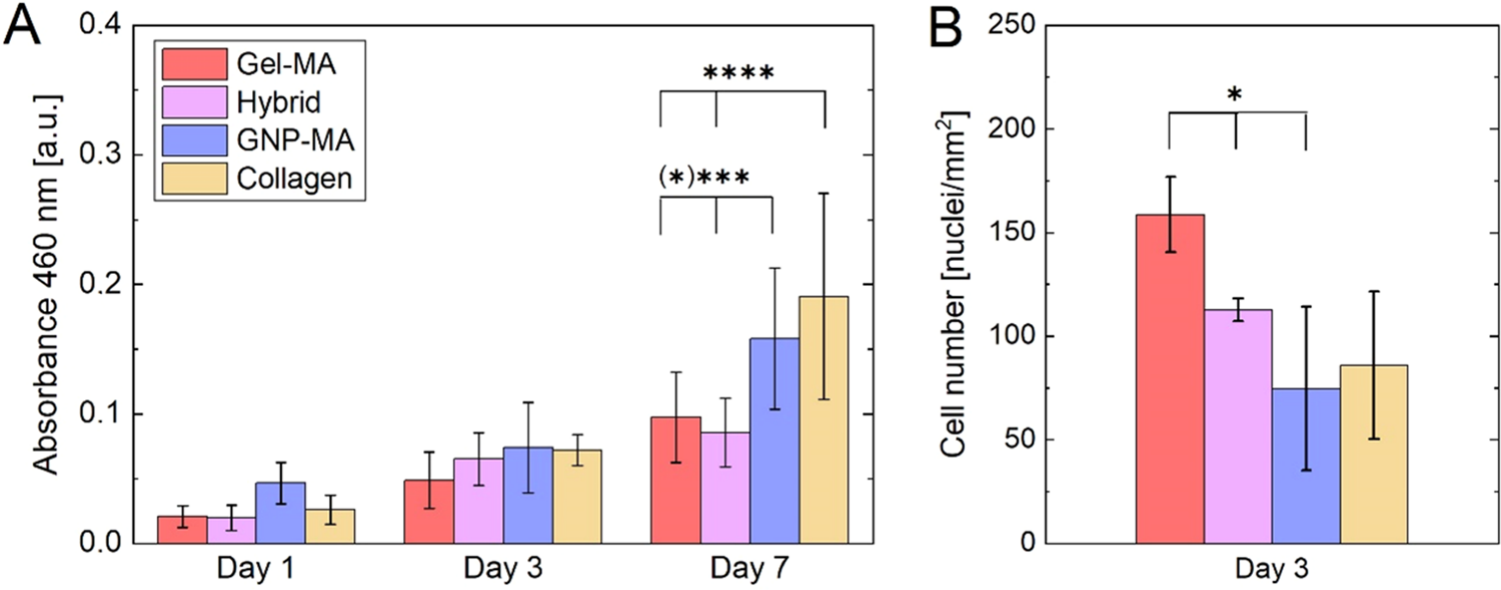
(A) Cell metabolic activity of murine pre-osteoblasts (MC3T3-E1) cultured in 2D on hydrogels at different time points determined by colorimetric CCK-8 assay. Data corresponds to mean and standard deviation with two-way ANOVA (*n* = 12, ***p < 0.001 and ****p < 0.0001). (B) Number of MC3T3-E1 cells after 3 days of 2D culture on hydrogels determined by confocal laser scanning microscopy image analysis (*n* = 3–5, *p < 0.05).

To determine the cytocompatibility of hydrogels and effects of their mechanical properties on cell proliferation and morphology, murine pre-osteoblasts (MC3T3-E1) were cultured in 2D on the three types of gelatin-based hydrogels as well as control collagen hydrogels. Figure 6A depicts the metabolic activity of cells cultured on the different hydrogels at different time points. Cell metabolic activity increased over time for all hydrogels indicating active cell proliferation, i.e., all hydrogels are cytocompatible. Importantly, the three hydrogels based on different gelatin building blocks, including the novel Hybrid and GNP-MA hydrogels, exhibited cell metabolic activity in a similar range as collagen, which is widely employed in cell culture.^4^ Statistically significant variations were found after 7 days of culture for collagen, which exhibited a higher metabolic activity than Gel-MA and Hybrid hydrogels (*p* < 0.0001), as well as GNP-MA, which exhibited a higher metabolic activity than Gel-MA (*p* < 0.0001) and Hybrid (*p* < 0.001) hydrogels. Analysis of cell nuclei per area from confocal laser scanning microscopy images (see Figure S2) indicates that the number of cells was significantly higher on Gel-MA compared to Hybrid and GNP-MA hydrogels (Figure 6B, *p* < 0.5), suggesting that the higher metabolic activity on GNP-MA and collagen hydrogels derives from increased cellular stress rather than higher cell proliferation. Hence, cell proliferation of MC3T3-E1 cells was higher on stiff hydrogels as previously reported.^64,65^

**Figure 7:**
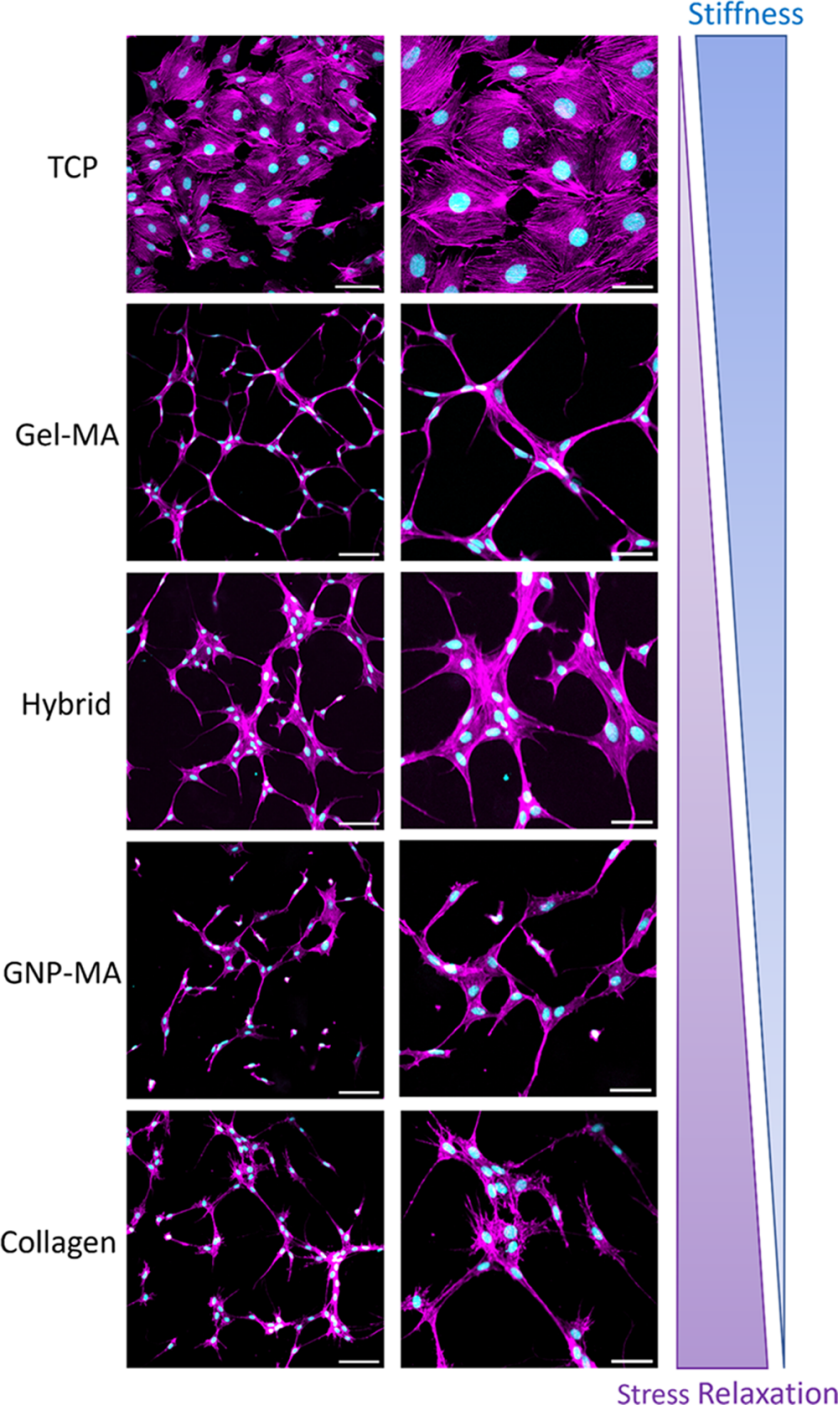
Confocal laser scanning microscopy images of murine pre-osteoblasts (MC3T3-E1) after 3 days of 2D culture on tissue culture-treated plastic (TCP) and different hydrogels with staining for nuclei (Hoechst, cyan) and F-actin (phalloidin, magenta). Scale bars correspond to 100 μm (left) and 50 μm (right).

The effect of hydrogel type and mechanical properties on cell morphology was determined by confocal laser scanning microscopy with fluorescent staining for cell nuclei and F-actin, as shown in Figure 7. The murine pre-osteoblasts (MC3T3-E1) were cultured for 3 days on gelatin-based hydrogels, control collagen, as well as tissue culture-treated plastic (TCP) as a reference for conventional cell culture on a solid substrate. On TCP, MC3T3-E1 cells exhibited a uniformly extended polygonal morphology, as typically observed for MC3T3-E1 cells cultured on solid substrates.^66,67^ In contrast, on all gelatin and collagen hydrogels the cells were more extended with pronounced protrusions. This change in cell morphology is likely induced by the presence of cell-binding motifs in gelatin and collagen hydrogels such as RGD-sequences which facilitate cell adherence and spreading that are not present on TCP. Wang et al.^68^ previously demonstrated that MC3T3-E1 cells remain polygonal on hydrogels in the absence of cell adhesion ligands, but adopt an extended morphology once adhesion ligands are introduced into the hydrogels. Deng et al.^69^ further confirmed that MC3T3-E1 cells do not spread below a given density of cell-binding motifs. Technically, all gelatin-based hydrogels contained similar amounts of adhesion ligands as they were prepared from the same type and solid content of gelatin. However, it is possible that the modification with methacryloyl groups or aggregation into particles affected the cellular availability of adhesion ligands.

Regarding hydrogel mechanical properties, we observed a trend toward more extended cell morphology, defined protrusions, and interconnectivity for stiffer hydrogels. MC3T3-E1 cells adopted the most extended morphologies with defined protrusions and characteristically thin cell bodies on the stiffest Gel-MA hydrogel. On Hybrid hydrogels, cells were less extended and more clustered. On GNP-MA hydrogels, cells were even less extended, and several isolated cells were apparent. Ultimately, on the softest collagen hydrogels, cells formed multiple short and undirected protrusions. Although it is known that pre-osteoblastic cells show faster proliferation on stiff hydrogels,^64,65^ the effect of hydrogel mechanical properties on cell morphology has not been investigated for this cell type to our knowledge. Our results suggest that MC3T3-E1 cells adopt a more extended shape with defined protrusions on stiffer hydrogels. Hence, hydrogels that are stiffer than commonly used collagen such as the present gelatin hydrogels may be preferable for a more natural cell spreading of cell types that prefer stiff substrates such as MC3T3-E1. Bauer et al.^15^ also found a more extended structure on stiffer hydrogels for murine myoblasts. It is noteworthy that the opposite behavior is generally observed for mesenchymal stromal cells, i.e., extended cell shapes on soft and polygonal shapes on stiff substrates.^70,71^ Several authors have recently also reported more pronounced cell spreading for hydrogels with increased stress relaxation.^15,17,18^ However, those hydrogels were usually designed to exhibit variable stress relaxation at constant stiffness. We did not observe the effects of stress relaxation despite considerable variations within the hydrogels (see Figures 3 and 4). We speculate that in the present case of culturing MC3T3-E1 cells which prefer stiff substrates the cell morphology is primarily dictated by hydrogel stiffness, and effects of stress relaxation might only be observed if hydrogels with matching stiffness, but variable stress relaxation are employed. As fast stress relaxation has been reported to favor osteogenic differentiation and bone healing,^18,19,21^ it is suggested that hydrogels with high stiffness as well as fast stress relaxation might be ideal for the culture of osteoblastic cells and for the regeneration of hard tissue. Besides mechanical properties, it is possible that cells respond to local variations in surface topography or crosslink density that depend on hydrogel architecture. We also noted that apparent hydrogel mechanics may vary depending on the mode and length scale of deformation, i.e., in macroscopic rheology vs. nanoindentation (Figures 2 and 5, respectively), stressing the need to consider hydrogel mechanics at a smaller scale to unravel the mechanical environment as perceived by cells.

## 4 Conclusions

We have established the modular fabrication of hydrogels by combining monolithic matrices and particulate building blocks to facilitate the formation of cytocompatible hydrogels with tailored mechanical properties. More specifically, we have designed three gelatin-based hydrogels with different physical architectures: (i) a fully monolithic gelatin methacryloyl (Gel-MA) hydrogel, (ii) a Hybrid hydrogel from 1:1 Gel-MA and gelatin nanoparticle (GNPs), and (iii) a fully particulate hydrogel based on methacryloyl-modified gelatin nanoparticles (GNP-MA) and GNPs. The three hydrogels were formulated at the same solid content and exhibited comparable storage moduli, but different stiffness and viscoelastic stress relaxation. The monolithic photo-crosslinked Gel-MA hydrogel was the stiffest hydrogel and showed limited stress relaxation. The incorporation of 50% nanoparticles by weight reduced the stiffness and slightly accelerated stress relaxation; however, the mechanical properties were still mostly dominated by the Gel-MA matrix. Hence, the incorporation of nanoparticles in covalently crosslinked hydrogels has a limited effect on macroscopic hydrogel properties; however, it may affect mechanical properties at smaller scales as revealed in nanoindentation experiments, indicating that the nanoparticles may be more readily displaced at a smaller. It remains to be determined if the incorporated particles may provide a more dynamic environment on a cellular scale and can promote cell activity or migration in 3D. The fully particulate hydrogels were even softer and exhibited accelerated stress relaxation, demonstrating the increased dynamicity of fully particulate hydrogels.

The three gelatin-based hydrogels were examined for 2D cell culture of murine pre-osteoblasts in comparison to collagen control hydrogels and tissue cultured plastic (TCP). The established collagen was softer and showed faster stress relaxation compared to the gelatin-based hydrogels. The gelatin hydrogels were cytocompatible and exhibited cell metabolic activity in a comparable range as collagen, with a trend to higher cell proliferation on stiffer hydrogels. The MC3T3-E1 cells adopted a considerably different elongated morphology with pronounced protrusions on all hydrogels compared to TCP due to the presence of cell adhesion ligands in gelatin and collagen hydrogels. Furthermore, we noted a trend to a more extended morphology and more pronounced protrusions on stiffer hydrogels, indicating that the present hydrogels may be more suitable for culturing cells with a preference for stiff substrate such as MC3T3-E1.

## Supporting Information Available

Photo-gelation, rheology, and stress relaxation of alternative formulations of modular hydrogels and
exemplary confocal laser scanning microscopy images used for detecting nuclei (PDF)
