# Supplementary material for: A Modular Platform for Cytocompatible Hydrogels with Tailored Mechanical Properties Based on Monolithic Matrices and Particulate Building Blocks
Source: Biomacromolecules. 2023 May 24;24(6):2755–65. doi: 10.1021/acs.biomac.3c00177 (PMC10265656; doi:10.1021/acs.biomac.3c00177)
Supplement: Supplementary file 1 — bm3c00177_si_001.pdf [file bm3c00177_si_001.pdf]

Supporting Information:

A modular platform for cytocompatible hydrogels with tailored mechanical properties based on monolithic matrices and particulate building blocks

Lea Andrée[1,a], Pascal Bertsch[1,a], Rong Wang[1], Malin Becker[2], Jeroen Leijten[2], Peter Fischer[3], Fang Yang[1] and Sander C. G. Leeuwenburgh[1,*]

[1]Radboud University Medical Center, Department of Dentistry – Regenerative Biomaterials, Radboud Institute for Molecular Life Sciences, Philips van Leydenlaan 25, 6525 EX Nijmegen, The Netherlands

[2]Leijten Laboratory, University of Twente, Department of Developmental BioEngineering, Faculty of Science and Technology, Technical Medical Centre, Drienerlolaan 5, 7522 NB Enschede, The Netherlands

[3]ETH Zurich, Department of Health Sciences and Technology, Institute for Food Nutrition and Health, Schmelzbergstrasse 7, 8092 Zurich, Switzerland

[a]Equal contribution

E-Mail: Sander.Leeuwenburgh@radboudumc.nl

Figure S1 shows the photo-gelation under blue light and rheology of alternative hydrogel formulations obtained from the different gelatin building blocks. Figure S1A depicts the photo-gelation of the Hybrid hydrogel employed in the main manuscript containing 1:1 Gel-MA and GNPs as well as an alternative formulation with higher nanoparticle fraction, i.e., 1:2 Gel-MA and GNPs, both at a solid content of 6 wt/v%. The hydrogel formulation with a higher fraction of GNPs exhibited a comparable G' after photo-gelation, however, the gelation kinetics were slower compared to the 1:1 formulation. The stress relaxation of the 1:2 formulation was still mostly dominated by the Gel-MA matrix and was limited to 20-30% (Figure S1B).

Figure S1C shows the photo-gelation of fully particulate hydrogels obtained from different ratios of anionic methacryloyl-modified GNP-MA and cationic GNPs. The highest G' was obtain by 1:2 GNP-MA (methacryloyl substitution degree (DS) = 60%) and GNPs, corresponding to the formulation investigated in the main manuscript. Employing GNP-MA with a lower degree of methacrylation of 40% had no considerable effect on photo-gelation or G'. On the other hand, using higher ratios of GNP-MA particles in 1:1 formulations resulted in lower G', particularly when using GNP-MA with 40% DS. Hence, despite using a higher fraction of photo-crosslinkable particles hydrogels with lower G' were attained, indicating that obtaining particle dispersions with neutral charge was more relevant to achieve high G' than incorporating more photo-crosslinkable methacryloyl-modified particles.

Figure S2 shows exemplary confocal laser scanning microscopy images of murine pre-osteoblasts (MC3T3-E1) after 3 days of 2D culture on the different gelatin hydrogels that were used for determining cell numbers (nuclei) per area.

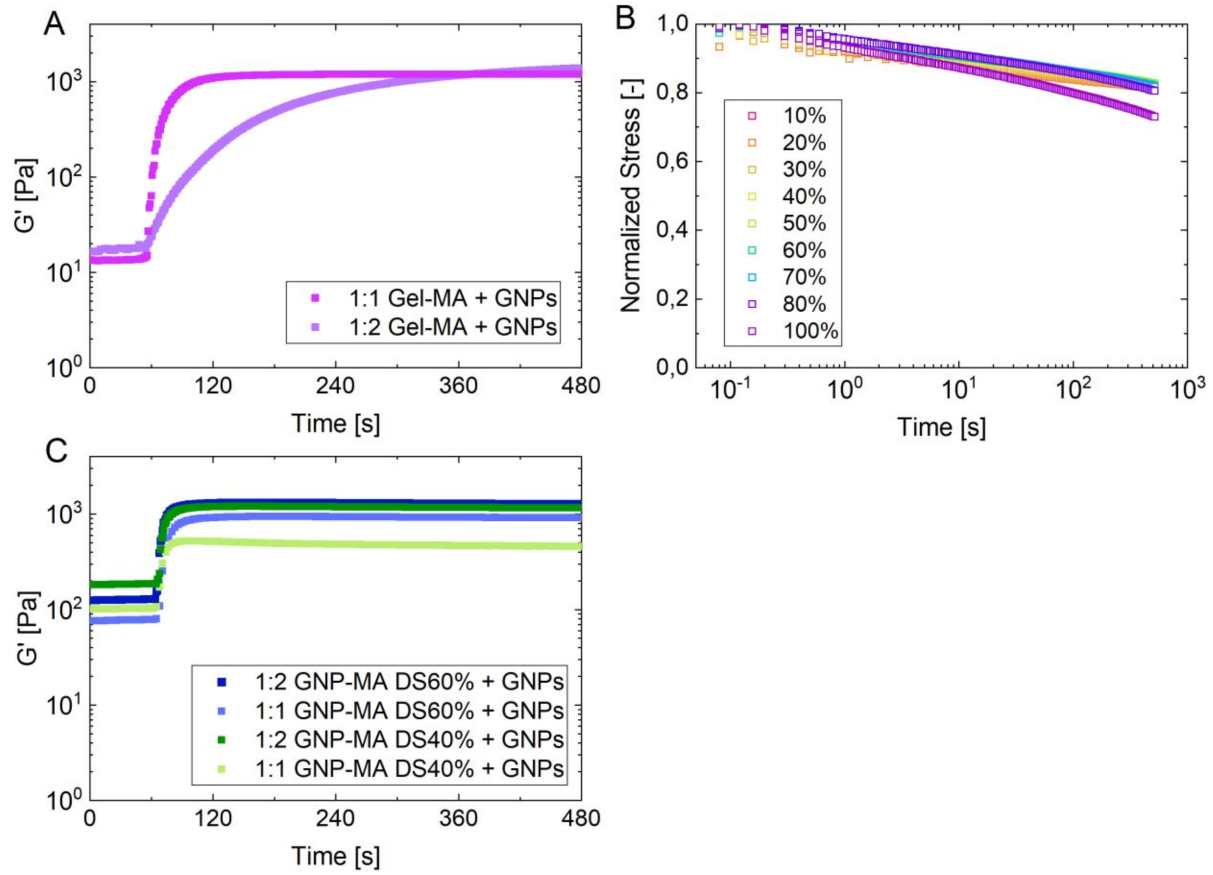

Figure S1: Rheological characterization of alternative 6 wt/v% gelatin-based hydrogel formulations showing (A) the gelation kinetics of Hybrid Gel-MA + GNP hydrogels at different ratios upon exposure to blue light expressed by increase in dynamic storage modulus G' and (B) the stress relaxation of 1:2 Gel-MA + GNPs Hybrid hydrogels. (C) Gelation kinetics of fully particulate hydrogels at different ratios of anionic methacryloyl-modified GNP-MA with varying degree of substitution and cationic GNPs.

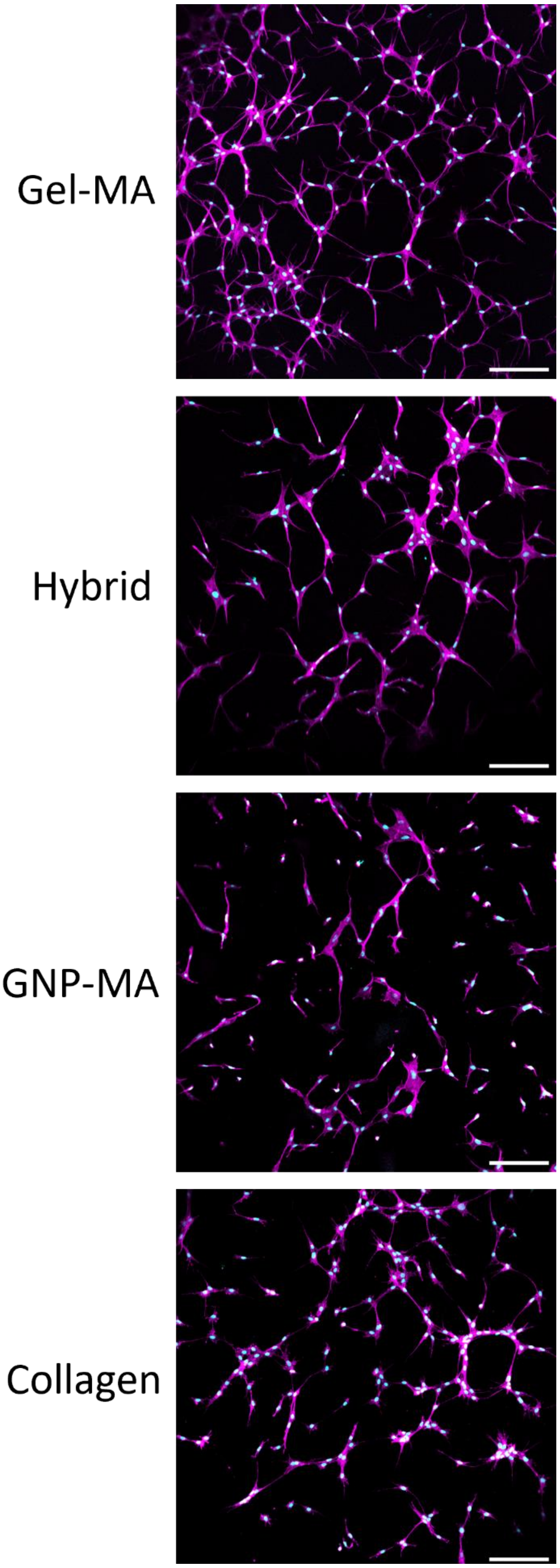

Figure S2: Confocal laser scanning microscopy images of murine pre-osteoblasts (MC3T3-E1) after 3 days of 2D culture on different hydrogels with staining for nuclei (Hoechst, cyan) and F-actin (phalloidin, magenta). Scale bars correspond to 200 µm.
